# Teachers’ Relationship Closeness with Students as a Resource for Teacher Wellbeing: A Response Surface Analytical Approach

**DOI:** 10.3389/fpsyg.2015.01949

**Published:** 2015-12-23

**Authors:** Anne Milatz, Marko Lüftenegger, Barbara Schober

**Affiliations:** Faculty of Psychology, University of ViennaVienna, Austria

**Keywords:** elementary teacher, burnout, student-teacher relationships, attachment security, response surface analysis

## Abstract

Teachers’ relationship quality with students has been argued to be an important source of teacher wellbeing. Thus, the current study aimed to investigate to what extent teachers’ relationship closeness toward students, combined with attachment security is a resource protecting against teacher burnout. Eighty-three elementary school teachers reported on their most and least attached student’s relationship closeness, their attachment security and levels of burnout, as measured by emotional exhaustion, depersonalization and personal accomplishment. Response surface analysis (RSA), enabling researchers to investigate the effect of congruence/incongruence of two predictors on an outcome, revealed that teachers’ depersonalization and emotional exhaustion were lowest when they developed homogenous close relationships toward the students within their classroom and when teachers in general made congruent relationship experiences. No RSA model could be specified for personal accomplishment, even though a correlational analysis revealed that increasing closeness with students fostered teachers’ personal accomplishment. Teachers’ secure attachment experiences were not directly related to burnout, but enhanced their capability to establish close relationships toward their students. Findings suggest that teachers’ relationships toward students are a resource for the teacher’s wellbeing, which highlights once again the importance of student–teacher relationships in education.

## Introduction

In the last decade serious concerns about teachers’ emotional wellbeing has been expressed repetitively. Statistics outline that up to 30% of the teachers are affected by burnout or psychological ill-being (Körner, 2003; Schaufeli and Bakker, 2004; Johnson et al., 2005; Hakanen et al., 2006; Unterbrink et al., 2007; Griebler, 2011; Schaarschmidt and Kieschke, 2013). In line with these statistics, being a teacher was rated as one of the most stressful jobs, as it is interpersonally and emotionally highly demanding (Johnson et al., 2005; O’Connor, 2008; Pyhältö et al., 2011).

Interpersonal relationships, for example with students, can be demanding and draining but can also be an important source of enjoyment and reward in a teacher’s daily life. Thus, the current paper mainly aims to provide empirical evidence to indicate the extent to which positive aspects of relational exchange with students, as reflected in relationship closeness, lead to decreased levels of burnout.

### Burnout – an Emotional and Relational Problem

The concept burnout was first introduced to empirical research as a human worker syndrome of emotional exhaustion, depersonalization and reduced personal accomplishment, which, until now, has been a widely accepted operationalization (Maslach and Jackson, 1981; Schaufeli et al., 2009). Emotional exhaustion, which refers to feelings of being psychologically drained and depleted, as well as depersonalization, having a cynical and negative attitude toward work and students, which results in distancing or uncaring reactions toward others, are seen as the core components of burnout (Schaufeli, 2003). The third component, reduced personal accomplishment, has been controversially discussed as being more of a personal characteristic than a central burnout component as it refers to feelings of competence and successful achievement and accordingly is most strongly positively related to feelings of self-efficacy (Schaufeli and Bakker, 2004; Alarcon et al., 2009).

Burnout develops when work becomes unpleasant, unfulfilling and unrewarding. When the negatives outweigh the positives it goes along with severe consequences for the individual such as negative job attitudes, illness-related consequences, and low organizational performance (Schaufeli and Enzmann, 1998; Rudow, 1999; Schaufeli, 2003; Hakanen et al., 2006; Schaufeli et al., 2009). In consequence, students are also affected as teachers ill-being was associated with lower educational quality and lower student achievements (Klusmann et al., 2006; McLean and Connor, 2015).

Consequently, efforts have been made to identify organizational, individual and interpersonal determinants of burnout. Interpersonal factors, most importantly teachers’ perception of student disruptive behavior, inattentiveness or perceived lack of respect of the students were identified as crucial determinants of burnout (e.g., Friedman, 1995; Hastings and Bham, 2003; Evers et al., 2004; Kokkinos, 2007). Positive aspects of social exchange have been less frequently focused on. However, students’ desirable social behavior, as well as social support from colleagues, has been shown to diminish teachers’ burnout (Friedman, 1995; Hastings and Bham, 2003; Schaufeli and Bakker, 2004; Hakanen et al., 2006).

Thus, it is not surprising that teachers’ burnout is widely accepted as a relational problem in which emotions and cognitions play a crucial role (Schaufeli and Enzmann, 1998; Rudow, 1999; Schaufeli, 2003; Montgomery and Rupp, 2005; Zembylas and Schutz; 2009). It has been shown that teachers’ judgments of student disruptive behavior, for example whether student behavior was perceived as a hindrance to their own goals, lead to unpleasant emotions which in turn foster burnout (Chang, 2013). Accordingly, the theoretical model of teacher burnout and emotions suggests that habitual patterns of teachers’ judgments lead to repeatedly experienced emotions which are responsible for teachers’ wellbeing (Chang, 2009; Chang and Davis, 2009). Along the same line of research, teachers’ increased anger, but also decreased enjoyment, was associated with an increased level of teacher’s emotional exhaustion (Keller et al., 2014).

### Student-Teacher Relationships – A Source of Teachers’ Daily Emotions and Cognitions

Teachers’ relationship experiences with students were argued to be an important daily source of teacher emotion and cognition, potentially affecting a teacher’s wellbeing (Spilt et al., 2011). In a comprehensive review the authors outline, among other aspects, that (1) within the framework of self-determination theory (=SDT, Deci and Ryan, 2000) it can be assumed that teachers have a need for relatedness and therefore student–teacher relationship quality plays a crucial role. Relatedness is one central basic need aside from autonomy and competence, which fosters sustainable and self-determined motivation. It is argued that those motivators might not only affect students’ learning, but also adult learning and most importantly their working process (Hakanen et al., 2006; Spilt et al., 2011). Furthermore, (2) within the attachment theoretical framework it was assumed that teachers’ relationship experiences with students, internalized in representational models, guide teacher’s daily emotions and cognitions in the classroom as it includes information about the relationship, the other and the self. Those relationship representations were suggested to mediate or moderate how a teacher judges a student’s daily behavior, leading to positive or negative emotions, which in turn contribute to teachers’ wellbeing (Spilt et al., 2011; Chang, 2013). To our knowledge, empirical evidence linking student-teacher relationships with teacher burnout has not been featured in the literature thus far.

#### Student–Teacher Relationship Research in Elementary School

Empirical research of student–teacher relationships within the attachment theoretical framework is based on the assumption that a teacher is a significant person in a student’s life and vice versa. Similarly, to parent–child relationships, the teacher serves as a secure base, which fosters students’ exploration and assistance in learning, especially in early and elementary education (Hamilton and Howes, 1992; Pianta et al., 2003; Schuengel, 2012; Verschueren and Koomen, 2012).

It is not surprising that relationships toward students have been shown to be among the top ten predictors for students’ academic outcomes in mega-analytic work including over 800 meta-analyses (Hattie, 2009) and thus are of specific interest in developmental as well as educational research. Nevertheless, research on STR within attachment perspective mostly focuses on the effects on students and not on the teacher. Until now, conflict-ridden relationships with students have been shown to be associated with teachers’ self-reported depression and lower self-efficacy (Hamre et al., 2008); whereas close relationships fostered teachers’ self-efficacy (Mashburn et al., 2006). The present research aims to increase our understanding how student–teacher relationships influences the teachers’ wellbeing and how a teachers’ attachment characteristics determine the teacher’s relationship quality.

Student–teacher relationship quality is mostly described by the extent of closeness, conflict and dependency as captured with the widely used student-teacher relationship scale (STRS, Pianta, 2001; Pianta et al., 2003). Those items aim to reflect internalized emotions and cognitions about the relationship and have been shown to be important promoters of students’ adjustment processes to the school setting and students’ academic, behavioral and social outcomes (e.g., Davis, 2003; Baker, 2006; Roorda et al., 2011).

#### Relevance of Student–Teacher Relationship Closeness in a Teacher’s Life

Research focusing on teaching as an emotional process provided evidence that student–teacher relationships fulfill a teacher’s crucial need for relatedness: interview data revealed that meaningful relationships with students were experienced as an important source of positive emotions, reward and satisfaction and were also a crucial motivation to enter into and to stay in the teaching profession (Hargreaves, 2000; O’Connor, 2008). Accordingly, teachers ranked student–teacher relationships as the most important and most satisfying part of their work (Shann, 1998). Relational closeness was most prominent in elementary school and not in secondary school (Hargreaves, 2000). In an elementary school sample relational emotions can be expected to be most intense and most variable.

Further support for the assumption that student–teacher relationships cause meaningful emotions in a teacher’s life is gained from social support research. Social interaction, social support and social integration have been shown to be essential for general health and for the stress buffering system due to its anxiety reducing and reward relevant consequences (Ditzen and Heinrichs, 2014). Teachers who develop affectionate bonds with their students, who feel valued and rewarded by them and who have the feeling that this affective work deposits itself in effective learning for the students (Hargreaves, 2000) should feel supported. Thus, teachers’ wellbeing may profit from close relationships.

Moreover, the diversity or range of relationship quality which a teacher experiences might be even more important as it mirrors a teacher’s daily relational exchange more in depth. The relationship range might reflect a teacher’s daily reward and challenge with relational needs and expectations. Teacher beliefs, such as to care for all students equally (Spilt et al., 2011), potentially embedded in teachers implicit theories of their classroom relationships, might shape teachers’ emotions (Chang and Davis, 2009): teachers might gain positive outcomes when they develop homogenous high quality relationships toward their students (congruent close relationships) as teachers fulfill their educational expectations as well as their relational needs. In contrast, overall more distant relationships with students might be associated with highest levels of burnout (Hargreaves, 2000). Incongruent or discrepant relationship experiences with students reflected in a wide range of relational closeness, might lead to feelings of incompetence, being unable to satisfy their needs and demands as a teacher and thus is perceived as goal incongruent and negative in their valence. Motive incongruence has, for example, been shown to decrease positive valence and affective commitment (Shanock et al., 2010; Schönbrodt, 2015a). All in all it is suggested that discrepant or incongruent relationship experiences with students might have a negative effect but thus far it seems to be rather exploratory. This relationship range was captured by assessing the STRS for the most and the least attached students in their classroom.

### Importance of Relationship Closeness and Attachment Security

The primary attachment experiences of teachers such as with their mother might shape the process of how relational closeness toward students is experienced. This may be because secure adults have internalized that significant others react promptly, effectively and reliably to the individual needs and thus they are experts on how to use relationships and support effectively and positively (Collins and Feeney, 2004; Mikulincer et al., 2009). Secure adults, for example, displayed more sensitive reactions toward the significant other’s needs, more effective coping as well as more positive and less negative emotions compared to individuals with insecure attachment experiences (Priel and Shamai, 1995; Mikulincer and Florian, 1997; Mikulincer et al., 2001; Mikulincer and Shaver, 2007). In contrast, individuals with insecure or anxious strategies have internalized that the partner reacts insufficiently to their support request which increases insecurity and anxiety as well as fear of rejection.

In conclusion, teachers with anxious attachment might be more vulnerable to suffer from (students’) interpersonal rejection than teachers with more secure attachment experiences. Thus, anxious attachment in combination with a low quality relationship with the most attached student might lead to more negative emotional experiences and thus might increases the risk of suffering from emotional ill-being (Horppu and Ikonen-Varila, 2004; Spilt et al., 2011). On the contrary, secure individuals with a high quality relationship toward the most attached student might profit most regarding their wellbeing.

Similarly to prior considerations about incongruent relational experiences, a negative effect on wellbeing can be assumed based on findings of motive-incongruence (Shanock et al., 2010; Schönbrodt, 2015a). On the other hand it may also be plausible that incongruent attachment and relational closeness experiences with students might not be related to increased burnout as long as connectedness with the student is high even though attachment security is rather mid to low, because the daily emotional exchange with students might be more important and rewarding (Hargreaves, 2000; O’Connor, 2008).

Furthermore, from attachment research on adults we expected that teachers’ attachment experiences also impact a teacher’s capability to form close relationships (Collins and Feeney, 2004; Mikulincer and Shaver, 2007; Mikulincer et al., 2009). As far as we know, there is only one study providing evidence that pre-service teachers who experienced harsh *Parental Discipline*, an indicator in the Attachment History Questionnaire, were more likely to experience decreased relationship closeness toward students (Kesner, 2000).

In order to address the proposed research questions of how the relationship range with students as well as how the most significant student combined with attachment security impacts teachers wellbeing, some methodological considerations of how to investigate the combined effect of two predictors on a third outcome variable needs to be addressed. Thus, in the following section, response surface analysis (RSA) is introduced as a powerful and statistical elaborated way to investigate the combined effect of two predictor variables on an outcome (Edwards, 2002).

### Testing for Combined Effects of Attachment: Response Surface Analysis (RSA)

Difference scores as predictors reflecting congruence or discrepancy are of limited use because no effects of how each predictor contributes to the outcome can be estimated and thus researchers cannot derive whether one predictor is more important than the other. Moreover, the level of the predictors, such as extent of closeness of the most and least attached student, which is assumed to affect the outcome, cannot be considered. Thus, no so called mean level effect can be estimated. Another problematic issue is that scale equivalence of the two predictors is often not met or not possible to obtain. As a consequence, effect interpretation of difference scores is ambiguous and the possible research questions which can be addressed are restricted. A huge disadvantage of moderated regression analysis is that no effect of how the discrepancy of two predictors affects the outcome can be estimated, e.g., how increasing heterogeneity/range of teacher’s relationship closeness with the most and least attached students predicts burnout. This so called incongruence/congruence or fit-effect cannot be explored in a two-dimensional space as it is provided by conventional regression models. Furthermore, only linear relationships between outcome and predictors are tested and not quadratic effects (Shanock et al., 2010).

**FIGURE 1:**
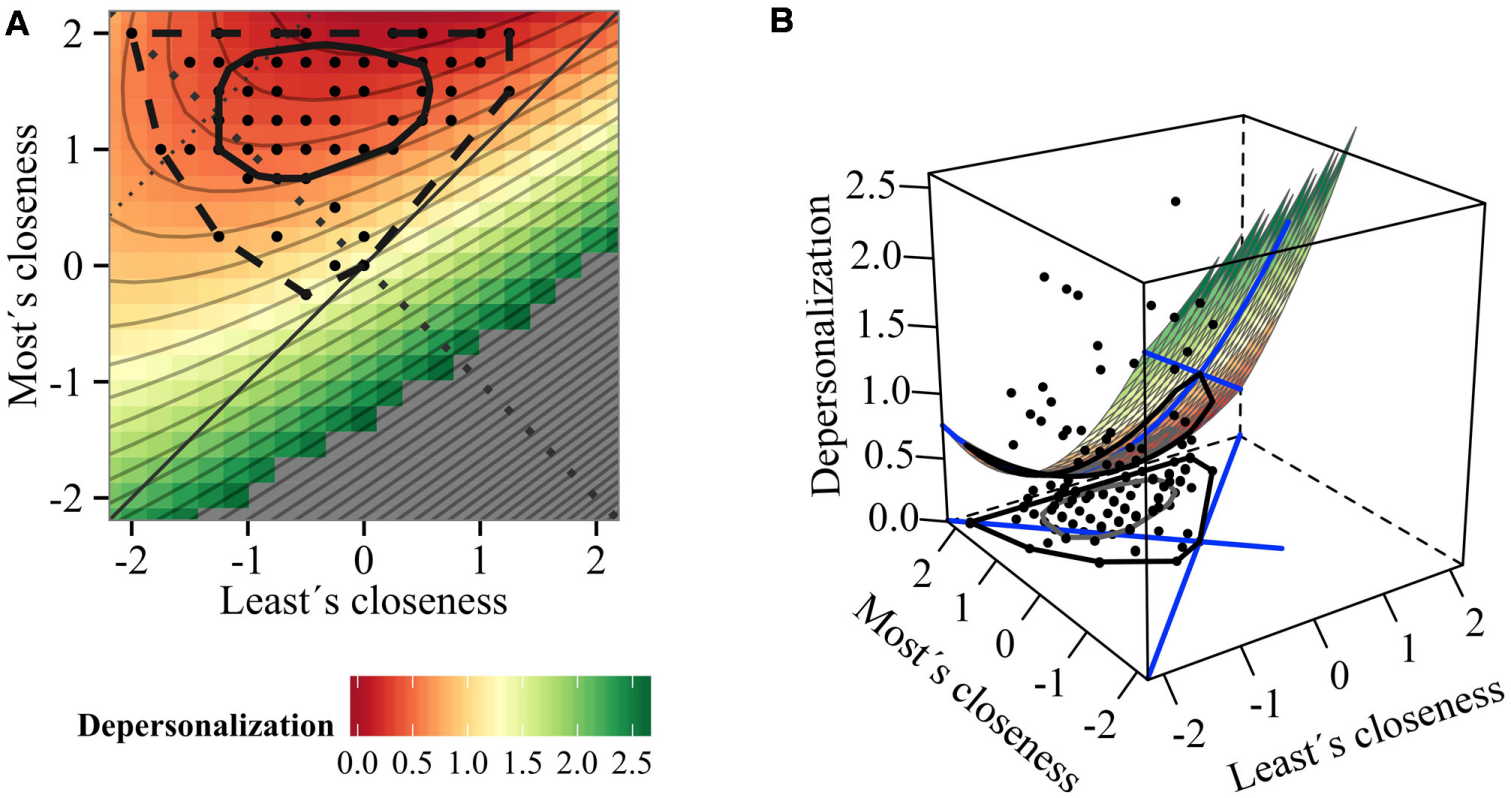
**Impact of teachers’ connectedness with students on depersonalization: (A) contour and (B) surface plot (Model 1)**.

**FIGURE 2:**
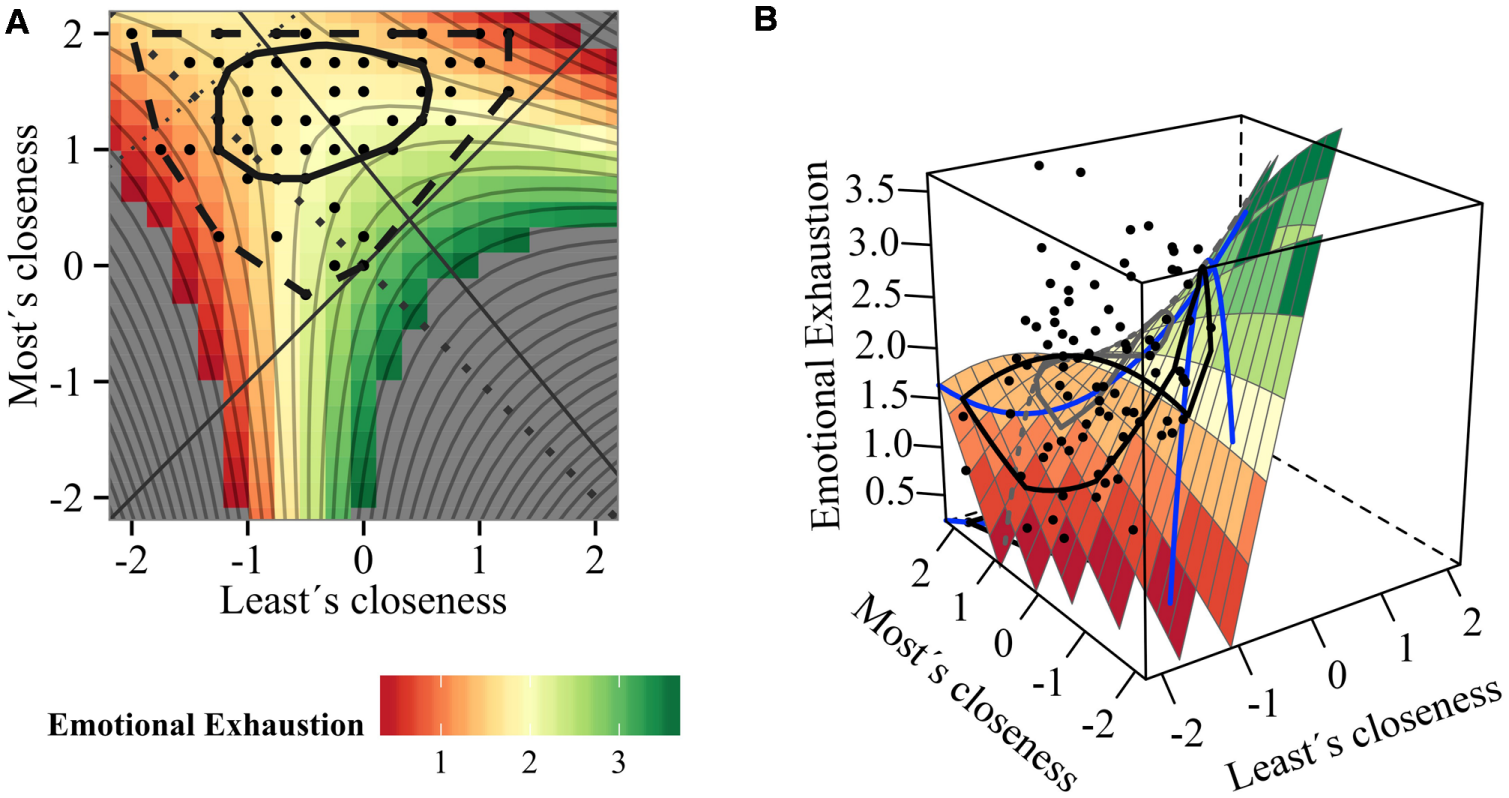
**Impact of teachers’ connectedness with students on emotional exhaustion: (A) contour and (B) surface plot (Model 1)**.

**FIGURE 3:**
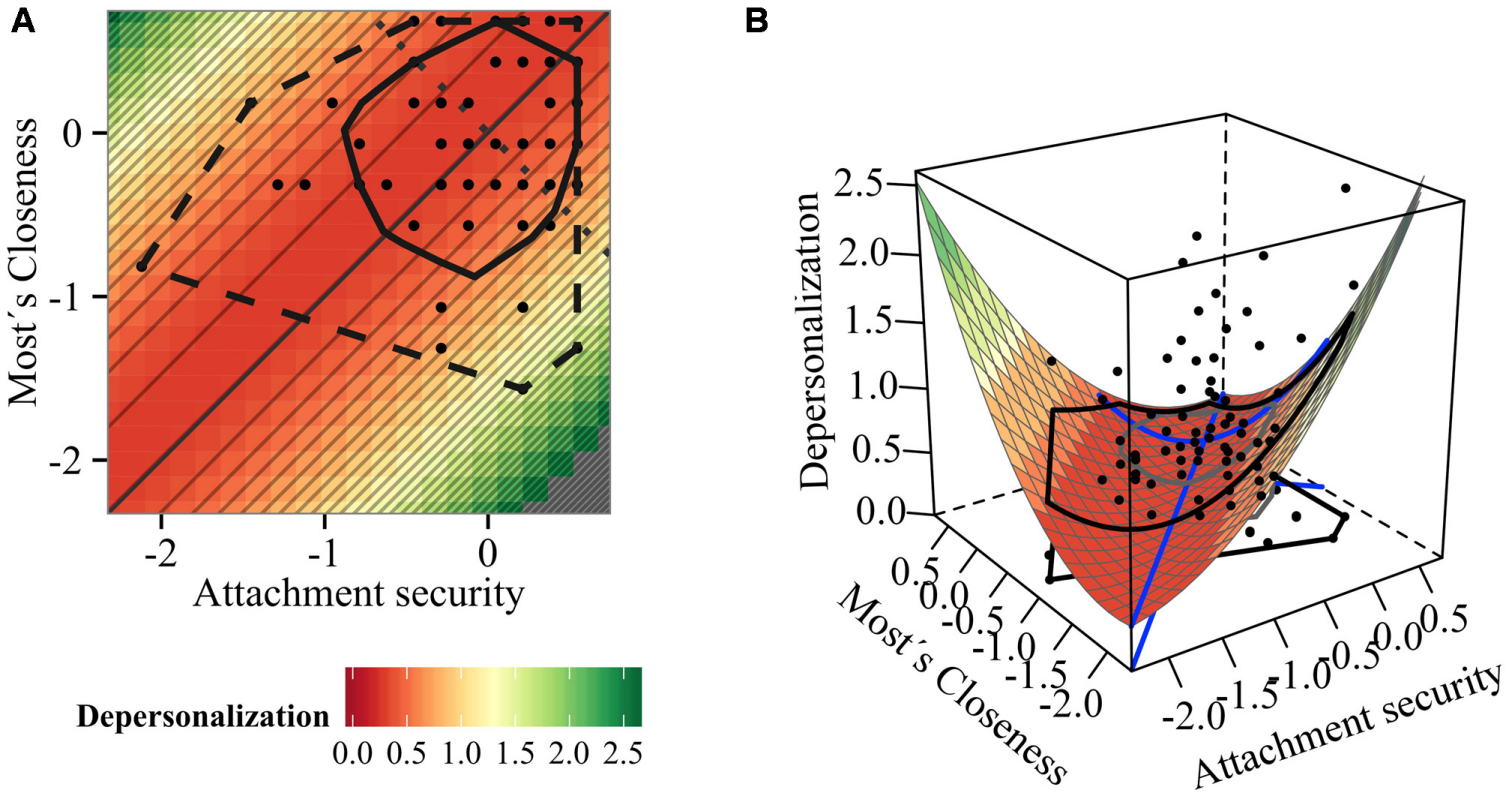
**Impact of attachment and student–teacher relationship closeness on depersonalization: (A) contour and (B) surface plot (Model 2)**.

**FIGURE 4:**
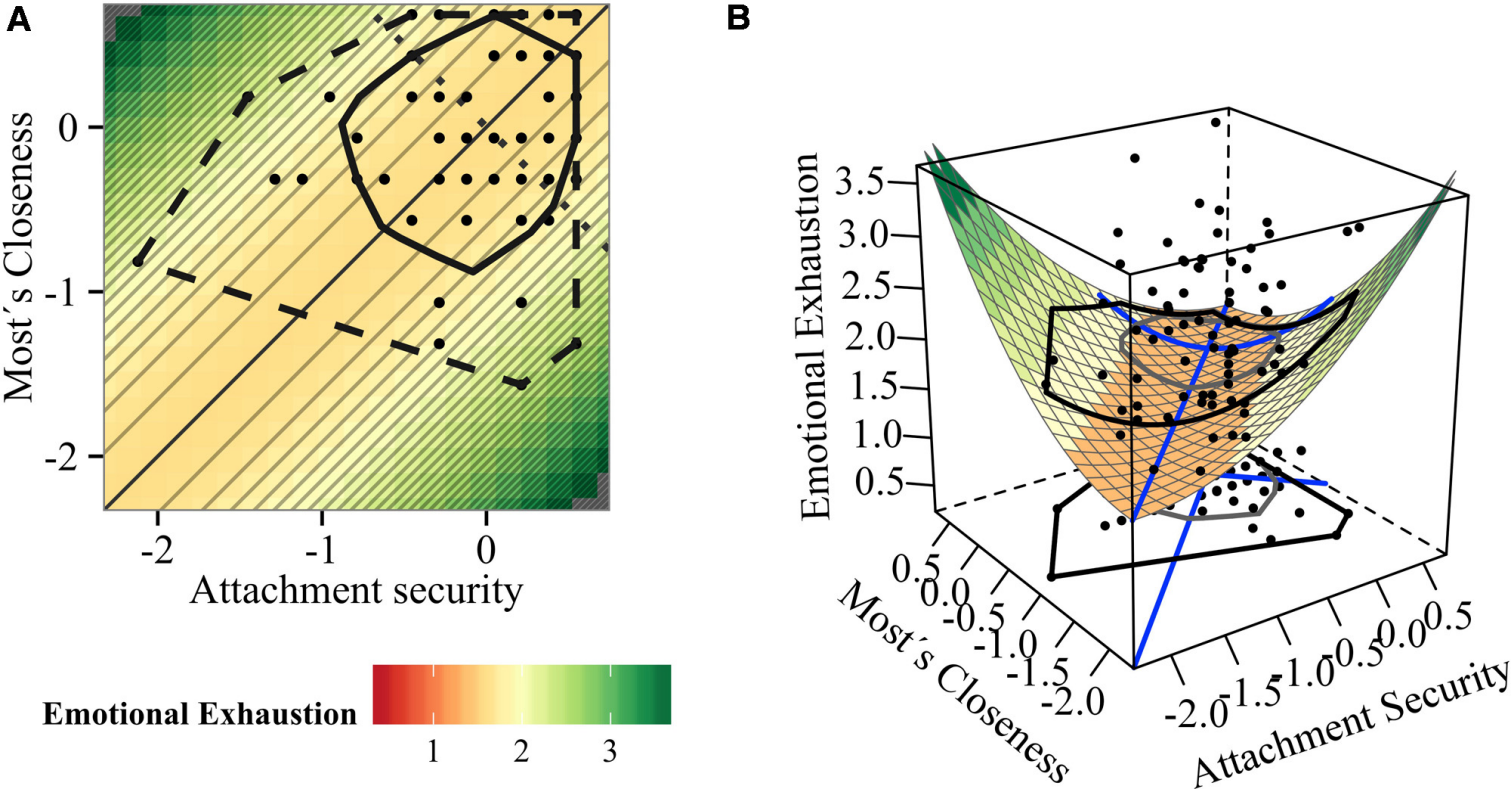
**Impact of attachment and student–teacher relationship closeness on emotional exhaustion: (A) contour and (B) surface plot (Model 2)**.

All those limitations of difference scores and regression models can be overcome when using RSAs. RSA models allow one to test whether congruence or discrepancy of two variables, such as most and least attached student’s closeness, is related to an outcome. By applying RSA models, researchers can overcome difficulties with traditional approaches such as using absolute or quadratic difference scores of the two predictors and by applying moderated regression models (Edwards, 2002; Shanock et al., 2010; Schönbrodt, 2015a). RSA models also allow to test for mean-level effects and fit-effects. Moreover, the results are illustrated in a three-dimensional surface plot and a respective contour plot (see **Figures 1**–**4**) which facilitate and guide interpretation. Until now, only commensurable predictor scales could be used, but recently introduced polynomial models, so-called Rising Ridge and Flat Ridge models, which are statistically simpler and nested within the full polynomial model, also allow for the inclusion of incommensurable predictor scales (Schönbrodt, 2015a). All in all, RSA models are a powerful way to explore level-effect and fit-effect hypotheses as aimed in the current paper (more details in the method section).

### Aims and Hypotheses of the Present Study

It can be concluded upon the presented theoretical and empirical considerations that accounting for student–teacher relationships and attachment experience might help to deepen the understanding of the relational nature of teachers’ burnout. Feelings of connectedness, support and joy as reflected in relationship closeness with the most attached student and in the teachers’ relational range of closeness, as well as the availability and trust in close relationships, such as with the mothers, might be protective factors for teacher burnout, which is recognized as a relational and emotional process (Mikulincer and Shaver, 2007; Chang and Davis, 2009; Zembylas and Schutz, 2009; Spilt et al., 2011; Chang, 2013). To our knowledge, empirical evidence investigating the role of teachers’ relationship quality toward students in combination with teachers’ attachment experiences for burnout is still lacking. Thus, the following aims addressing this gap in the literature motivated the current study:

First (Q1), we examined how teachers’ connectedness, especially the range of relational closeness toward the students affects teacher burnout. We assume lowest burnout levels when teachers in general develop homogenous close relationships toward their students (low range and high levels of closeness) and highest burnout levels when teachers experience homogenous low connectedness toward their students (low range and low levels of closeness). Also we explored whether relational incongruence negatively impacts teacher burnout.

Second, (Q2), we tested for the combined effect of teachers’ connectedness toward the most attached student and teachers’ attachment security, on burnout. We assume a high connectedness in combination with secure attachment experiences to be associated with the lowest burnout whereas attachment anxiety combined with low relational closeness with the student is assumed to be associated with the highest burnout levels.

Before addressing the main research questions, we aimed to explore intercorrelations of all scales. We assumed that the most attached student’s closeness and attachment security serve as protective factors against burnout. Furthermore, attachment security should be associated positively with the most attached student’s closeness.

## Materials and Methods

### Procedure and Sample

The sample is a convenience sample since teachers were contacted personally by student research assistants in the first quarter of 2011. The present study was conducted in compliance with ethical standards provided by the Austrian Federal Ministry of Health (Bundesministerium für Gesundheit, 1995) and the American Psychological Association [APA] (2010). Accordingly, prior to participation, teachers were informed about the goals of the study, its duration, procedure and the anonymity of their data by the respective student research assistants during the first appointment in school. Participation was voluntarily at any time. After informed consent was provided, teachers were interviewed individually (this data is not presented in the current paper). Afterward teachers were asked to fill in the questionnaires within 2 weeks, which were then collected personally by student research assistants.

Teachers were asked to fill in a paper–pencil questionnaire survey including sociodemographics, the Maslach Burnout Inventory (MBI, Enzmann and Kleiber, 1989), attachment security scale (Asendorpf et al., 1997) and the closeness scale of the STRS twice (Milatz et al., 2014). Teachers were instructed to select two target students from their current classroom – one student the teacher “feels most attached to” and one student she “feels least attached to.” Teachers were asked to report about age and gender of the respective student before filling in the STRS questionnaire for both target students.

The present study was conducted in compliance with ethical standards provided by the Austrian Federal Ministry of Health (Bundesministerium für Gesundheit, 1995) and the American Psychological Association [APA] (2010). Accordingly, prior to participation, teachers were informed about the goals of the research, duration, procedure and anonymity of their data, participation was voluntarily at any time and informed consent was provided. Data was collected and analyzed anonymously.

Eighty-three female elementary classroom teachers from first to fourth grade (*M* = 2.2, *SD* = 0.91) were involved in the present study. Teachers were engaged in 45 different elementary schools; whereas *n* = 65 teachers were from 31 Austrian schools and *n* = 18 teachers were from 13 German schools. On average, teachers were 36.4 years old (*SD* = 10.87) and responsible for 21.22 students (*SD* = 4.33) in their respective classrooms. The teachers spend on average 21.12 h (*SD* = 5.37) per week with their students in the classroom. Their total work load per week comprised on average 36.53 h (*SD* = 9.02). Work experience of the teachers ranged from 1 to 37 years (*M* = 12.43, *SD* = 11.04). Because each teacher reported on two students in their class, *N* = 166 students (*n* = 72 female) were also involved. On average, students were 7.94 years old (*SD* = 1.2).

### Measures

#### Burnout

A German version of the widely used MBI (Enzmann and Kleiber, 1989) comprising three facets and in total 22 items was applied. The scale assesses emotional strain and accomplishment by asking how often certain work-related emotions and cognitions are present. The items were evaluated on a 7-point Likert scale ranging from “never” to “daily.” High values in emotional exhaustion describe a feeling of being drained and of lacking emotional resources (*k* = 9, α = 0.83, e.g., “I feel burned out from my work.”). A high extent in depersonalization reveals a negative, indifferent and cynical attitude toward students and people in general (*k* = 5, α = 0.65, e.g., “I don’t really care what happens to some students.”). In contrast, high values in personal accomplishment describe feelings of appreciation, competence and subjective satisfaction with own performance (*k* = 8, α = 0.72, e.g., “I have accomplished many worthwhile things in this job.”).

#### Student–Teacher Relationship Quality

Relational closeness was assessed with the Student-Teacher Relationship Scale (Pianta, 2001), recently adjusted for the German context (Milatz et al., 2014). Closeness to most attached/least attached student describes the extent to which the interaction between teacher and student is characterized by reciprocal support, sympathy and warmth as well as open communication about feelings (*k* = 4; α = 0.66/0.73, e.g., “This child openly shares his/her feelings and experience with me.”). Items were rated on a 5-point Likert scale ranging from “1: definitely does not apply” to “5: definitely applies.”

#### Attachment Security

Attachment security toward the mother was assessed with a German attachment scale developed upon the prototypic descriptions of the secure and fearful attachment styles (Bartholomew and Horowitz, 1991; Asendorpf et al., 1997). The bipolar attachment security scale measures attachment security, for example, with the item “It is easy for me to become emotionally close to my mother” and attachment anxiety, for example with “I worry that I am not accepted by my mother” (*k* = 6; α = 0.75). Items are evaluated on a 5-point Likert scale ranging from “Not at all true” to “Completely true.” High values represent attachment security and low values attachment anxiety.

### Data Analysis Strategy

All analyses were conducted using the software R 3.1.2 (R Development Core Team, 2014). First, descriptive results such as mean and standard deviation and intercorrelations of all scales are presented.

To test for the combined effects of relationship and/or attachment experiences on burnout (Q1 and Q2), three sets of RSA models – one set for each of the three burnout variables – were run for each of the two research questions; thus in total there were six sets of RSA models. Those polynomial models were run and plotted within *RSA* R-package (Schönbrodt, 2015b). Each set of models tested for the best model among eight candidate models: full polynomial model, three Rising Ridge models (RR, SRR, SRRR), four Flat Ridge models (SQD, SSQD, SRSQD) as well as the null model (further details regarding the models in section “RSA Methodology”). Since a meaningful zero point is important, all scales were centered. Robust *SE*s, *p*-values and CIs are reported. Checking for multivariate outliers and violations of normality assumption of residuals via qqplot revealed no strong violations (Bollen and Jackman, 1985).

#### RSA Methodology

Since the application of RSA models is still rather rare in the literature, a short overview of the methodology is provided here. The general RSA model is a polynomial regression model of second order including two predictors *X* and *Y*, the interaction of both predictors (X*Y) and the squared terms of both predictors (*X*^2^ and *Y*^2^) resulting in the following regression equation: *Z* = *b*_0_ + *b*_1_*X* + *b*_2_*Y* + *b*_3_ *X*^2^ + *b*_4_ *XY* + *b*_5_*Y*^2^ + 𝜀. The regression coefficients cannot be interpreted in isolation, and surface parameter tests have been derived in order to test for congruence (level-effect) or discrepancy hypothesis (fit-effect; more details in “Interpretation of RSA Parameters”; Edwards, 2002; Shanock et al., 2010; Schönbrodt, 2015a).

Congruence or agreement means that both predictors are more or less on the same level, e.g., closeness scores of most and least attached child are the same in conditions of perfect agreement when *X* = *Y*. This line is called line of congruency (LOC) and is depicted in **Figure 1A** by the straight line. In other words, congruence of teachers’ relationship closeness would indicate that teachers have rather homogenous relationship experiences toward their students in the classroom, because most and least attached students closeness share a comparable level. Thus, the congruence hypothesis, based on the full polynomial model, tests linear (*a*_1_) and quadratic (*a*_2_) relationships of how the level of congruence is related to the outcome (level effect). An example thereof is whether lower levels of closeness of the most and least attached students are differently related to the outcome than higher levels of most and least attached student’s closeness (linear relationship).

Perpendicular to the line of congruence is the line of incongruence (LOIC) in which *X* = -*Y*, as depicted by the dotted line in **Figure 1A**: the higher the distance from the intersection of LOIC and LOC toward the corners, the higher the general extent of incongruence between both predictors. For example, teachers’ relationship experiences with their mothers and students become more discrepant as the relationship quality of the student gets better than relationship quality with the mother or vice versa. Thus, incongruence or discrepancy of two predictors can be generally observed in two directions *X* > *Y* and/or *Y* > *X^1^.* In conclusion, discrepancy hypothesis, based on the full polynomial model, tests whether the general extent of incongruence (quadratic effect: *a*_4_) and the direction of incongruence affects the outcome (linear effect: *a*_3_). For example, it can be investigated as to whether discrepant or incongruent relationship experiences of teachers with students and mothers have an impact on their wellbeing (fit-effect).

The Flat ridge models include additional constraints, allowing for a shift in the ridge (SSDQ model) and additionally a rotation of the ridge (SRSQD model). Rising Ridge models allow a down or upward tilt of the ridge (RR models), as well as an additional shift (SRR model) and an additional rotation (SRRR model). Compared to the full polynomial model, those new models are statistically simpler but allow for the testing of more complex relationships and thus statistical power to detect fit patterns is enhanced. For all those new models, a fit-effect can be computed as well as a mean-level effect for Rising Ridge models (more details see Schönbrodt, 2015a).

#### RSA Model Selection

In order to select the best fitting model among the candidate models, several widely applied fit statistics were used. The *comparative fit index* (CFI) a sample size unbiased statistic compared to χ^2^ statistics was used to evaluate the absolute model fit (Bentler and Bonett, 1980). CFI values ≥0.90 were considered to reveal acceptable model fit and values ≥0.95 were deemed good model fit according to widely accepted rules (Hu and Bentler, 1999). As a comparative model statistic the corrected *Akaike information criterion* (AICc) was used, lowest AIC values indicate the best model (Burnham et al., 2011). To compare models ΔAICc was computed; where values <2 reveal that models are more or less equivalent (Burnham et al., 2011; Symonds and Moussalli, 2011). Thus, in the result section only models with ΔAICc < 2 compared to best model among the candidates are reported and relevant for model selection. Model weights, which is the probability that the respective model is the best among candidates and evidence ratios, indicating how many times a model is more likely than the other, are also reported (Burnham and Anderson, 2002; Wagenmakers and Farrell, 2004). In order to evaluate the general impact of the model, *R*^2^ was evaluated as well as the general model significance test.

#### Interpretation of RSA Parameters

In order to test congruence or incongruence effects, so-called surface coefficients *a*_1_–*a*_4_ derived from the regression coefficients *b*_1_–*b*_5_ can be computed for the full polynomial model (Shanock et al., 2010). The *a*_1_ coefficient (*a*_1_ = *b*_1_ + *b*_2_), also called mean-level effect (*b_M_*), estimates the slope along LOC (linear relationship of the two predictors with the outcome). For example, a negative *a*_1_ coefficient would suggest that higher levels of most and least attached students’ closeness are related with a lower outcome compared to lower levels of students’ closeness scores. The *a*_2_ coefficient (*a*_2_ = *b*_3_ + *b*_4_ + *b*_5_) estimates whether there is a curve-linear effect along LOC. Thus, a significant *a*_2_ coefficient indicates that the two predictors are not linearly but curve-linearly related to the outcome. In general, a positive *a*_2_ suggests an upward curve, which means that higher and lower levels of congruency of the two predictors go along with an increase of the outcome. In contrast, a negative *a*_2_ represents a downward curve and thus lower and higher levels of congruency are related with a lower outcome.

The slope along LOIC is assessed with the *a*_3_ coefficient (*a*_3_ = *b*_1_ -*b*_2_). It is an indicator of how the direction of incongruence (*X* > *Y* or *Y* > *X*) is related to the outcome. A significant positive *a*_3_ coefficient would indicate that when *X* > *Y* the outcome is higher compared to when *Y* > *X*. Similar to the LOC, a curvilinear effect can also be estimated for the LOIC by the *a*_4_ coefficient (*a*_4_ = *b*_3_-*b*_4_ + *b*_5_). This effect coefficient describes how far the degree of incongruence/range of relationships might be related with an increase (positive coefficient, upward curve) or a decrease (negative coefficient, downward curve) of the outcome. The *a*_4_ coefficient is also used for testing the fit effect for SRR, SQD, and SSQD model.

These tests for the dependency of burnout levels on two predictors can be best understood by exploring the response surface visually. Three dimensional plots showing the whole surface in the respective three-dimensional space and contour plots showing the most important part of the surface are provided. Colors depict the respective burnout level as indicated by the legend. It is important to note that only areas for which data exists should be interpreted. Thus, all plots include the scatter plots projected on the floor and a so called bagplot with an outer circle including 100% of the data points without outliers and the inner circle comprising 50% (Schönbrodt, 2015a).

## Results

### Descriptives and Inter-correlations of the Burnout and Relationship Scales

Means, standard deviations and inter-correlations for all burnout and relationship scales are depicted in **Table 1**. Means reveal that teachers in the current study show a rather low extent of burnout and a relatively high attachment security.

**Table 1:** Means, standard deviations, and interrelations of burnout and relationship scales.

Measure	*M (SD)*	1	2	3	4	5
(1) Emotional exhaustion	1.71 (0.82)	–				
(2) Depersonalization	0.45 (0.58)	0.38ˆ***	–			
(3) Personal accomplishment	4.75 (0.77)	-0.25ˆ*	-0.33ˆ**	–		
(4) Closeness_mostattachedstudent_	4.32 (0.52)	-0.20ˆ*	-0.37ˆ***	0.20ˆ*	–	
(5) Closeness_leastattachedstudent_	2.51 (0.75)	0.07	-0.04	0.03	0.08	–
(6) Attachment security	4.45 (0.55)	-0.11	-0.07	-0.05	0.20ˆ*	-0.04

*p < 0.05, **p < 0.01, ***p < 0.001.

Comparing the relationship quality of the most and least attached student revealed that all of the most attached students received higher values in closeness than their counterparts and closeness scores differed significantly [*t*(146.75) = 18.04, *p* < 0.001, *d* = 2.8]. Thus, it can be assumed that teachers applied the special instruction given for the STRS correctly, because most attached students were expected to receive higher relationship closeness. Regarding gender, the most attached student was 63.9% female (*n* = 53) and the least attached student was 77.1% male (*n* = 64). No differences were found regarding age between both students [*M*_most attached student_ = 7.92, *SD* = 1.24 vs. *M*_least attached student_ = 7.96, *SD* = 1.2, *t*(163.85) = -0.25, *p* = 0.799].

The strongest associations between burnout and relationship scales could be found between teachers’ most attached student’s closeness and depersonalization (*r* = -0.37, *p* < 0.001) as well as emotional exhaustion (*r* = -0.20, *p* < 0.05) and personal accomplishment (*r* = 0.20, *p* < 0.05). Those correlations reveal that teachers with high connectedness toward the most attached student describe themselves less depersonalized, exhausted and more effective. Regarding attachment security, no association with burnout could be found, but attachment security was positively related with closeness of the most attached student in classroom (*r* = 0.20, *p* < 0.05). Thus, the more secure and trustful teachers experienced the relationship with their mothers to be, the closer the relationships that teachers develop with their most significant student in class.

### Q1: Impact of Teachers’ Connectedness with Students, on Burnout

In the first set of RSAs, the impact of the teachers’ relationship range on the respective burnout scales was modeled. Closeness scores of the least attached student were entered as *X* variable and the most attached student’s closeness as *Y* variable.

For Depersonalization, the best model according to AIC was the SRR, followed by the SRSQD, the only model which was equally plausible with a ΔAICc < 2 (see detailed model indices in **Table 2**). Model weight was highest for the SRR model with 0.49. SRSQD revealed a lower model weight of 0.22 and was 2.26 times less likely than SRR. Due to this evidence we chose the SRR model, which also showed a good relative model fit according to a CFI of 1. Moreover, the SRR revealed an *R*^2^ of.175, which was statistically significant (*p* = 0.002) and in total explained 1.6% more variance than the SRSQD model.

**Table 2:** Candidate RSA models and their goodness-of-fit indicators.

Model	*k*	AICc	ΔAICc	Model weight	Evidence ratio	CFI	*R*^2^	*p* model	*R*^2^*_adj_*
**Model 1: Impact of least^∗^most attached student–teacher relationship closeness on burnout**					
Depersonalization									
**SRR**	**5**	**707.50**	**–**	**0.49**	**–**	**1.00**	**0.175**	**<0.002**	**0.143**
SRSQD	6	709.13	1.63	0.22	2.26	1.00	0.159	<0.003	0.127
Emotional exhaustion									
**Full**	**7**	**770.29**	**–**	**0.44**	**–**	**1.00**	**0.176**	**0.009**	**0.122**
SRRR	6	770.50	0.20	0.39	1.11	0.86	0.151	0.011	0.107
**Model 2: Impact of attachment security^∗^most attached student-teacher relationship closeness on burnout**						
Depersonalization									
**SSQD**	**4**	**527.59**	**–**	**0.26**	**–**	**0.98**	**0.159**	**<0.001**	**0.138**
SRR	5	527.82	0.23	0.23	1.12	1.00	0.178	0.001	0.147
SQD	3	528.62	1.03	0.15	1.68	0.81	0.126	<0.001	0.116
SRSQD	5	529.22	1.63	0.11	2.26	0.94	0.164	0.002	0.132
SRRR	6	529.50	1.91	0.10	2.60	1.00	0.183	0.003	0.141
RR	4	529.56	1.97	0.10	2.67	0.82	0.139	0.003	0.117
Emotional exhaustion									
**SQD**	**3**	**594.34**	**–**	**0.37**	**–**	**1.00**	**0.046**	**0.051**	**0.034**
RR	4	595.62	1.28	0.20	1.89	1	0.055	0.102	0.031
Null	2	596.18	1.85	0.15	2.52	0	0.0	0.146	0.0

*Candidate models with ΔAICc < 2 compared to best model are depicted, selected models printed in bold, k = number of parameters, AICc, corrected Akaike information criterion; Evidence ratio, ratio of model weights of the best model compared to each of the other models; CFI, comparative fit index; R^2^, variance explained by the model; R^2^adj., adjusted; SRRR, shifted and rotated rising ridge model; SRR, shifted rising ridge model; RR, rising ridge model; SRSQD, shifted and rotated rising ridge model; SSQD, shifted squared difference model; SQD, squared difference model, Full, full model, Null, null model.*

**Figures 1A,B** illustrates the contour and 3d plot of the SRR model. Parameter estimates are printed in **Table 3**. The significant *a*_1_ or mean level coefficient (*a*_1_/*bm* = -0.404, *p* = 0.004) as well as the plots, reveal that teachers who establish rather homogenous high quality relationships within the classroom report less depersonalization than teachers who developed homogenous lower quality relationships toward their students. The incongruence/fit effect *a*_4_ tended toward significance (*a*_4_ = 0.466, *p* = 0.084), indicating that increasing heterogeneity in relationship quality goes along with increased depersonalization. Thus, as relationship experiences with students become more incongruent, the teachers feel more depersonalized, which means that teachers with rather heterogeneous relationship experiences in their classrooms tend to elicit more cold and impersonal responses than teachers who develop more similar or homogenous relationships toward their students. The plots further show that the most attached student’s relationship quality is apparently the stronger predictor because depersonalization increases most strongly in the contour plot [1(A)] as the relationship quality of the most attached student decreases (surface gets more yellowish when an imagined horizontal line goes down).

**Table 3:** Response surface analysis (RSA) Coefficients Model 1: impact of teacher’s connectedness with students.

Model	Estimate	Robust *SE*	95% CI (lower)	95% CI (upper)	*p*
**Depersonalization – SRR model**					
*b*_1_	0.432	0.263	-0.142	1.111	0.129
*b*_2_	-0.836	0.298	-1.515	-0.129	0.025
*b*_3_	0.117	0.067	-0.018	0.326	0.084
*b*_4_	-0.233	0.135	-0.651	0.035	0.084
*b*_5_	0.117	0.067	-0.018	0.326	0.084
*b_M_ /a*_1_	-0.404	0.141	-0.675	-0.115	0.004
*a*_4_	0.466	0.270	-0.071	1.302	0.084
C	-2.719	0.578	-6.671	-0.839	0.037
**Emotional exhaustion – full model**					
*b*_1_	1.195	0.381	0.447	1.943	0.002
*b*_2_	-0.531	0.592	-1.691	0.628	0.369
*b*_3_	-0.203	0.114	-0.428	-0.021	0.076
*b*_4_	-0.798	0.242	-1.273	-0.324	0.001
*b*_5_	-0.041	0.258	-0.547	0.465	0.873
*a*_1_	0.664	0.730	-0.768	2.096	0.363
*a*_2_	-1.043	0.422	-1.871	-0.214	0.013
*a*_3_	1.726	0.676	0.400	3.053	0.010
*a*_4_	0.554	0.307	-0.047	1.157	0.071

*SRR, shifted rising ridge model.*

The same procedure was repeated for the outcome variable Emotional Exhaustion. The full model was the best model and equally plausible was the SRRR model with a ΔAICc < 2 (**Table 2**). Because the SRRR model was not only inferior regarding model weight and evidence ratio, but also showed inacceptable model fit with a CFI of.86, we chose the full model. The full model revealed good model fit with a CFI of 1, explained most variance with an *R*^2^ of 0.176 and was statistically significant (*p* = 0.009).

The surface coefficients *a*_2_ and *a*_3_ were significant and *a*_4_ tended toward significance; whereas *a*_1_ was insignificant (**Table 3**). The negative *a*_2_ coefficient (*a*_2_ = -1.04, *p* = 0.013) suggests a curvilinear association, specifically a downward curve along the line of congruence (see **Figures 2A,B**). This means that teachers who develop rather homogenous high or low quality relations with their students are less emotionally exhausted than teachers who develop mid-range quality relationships. This can be also seen in **Figure 2B** in the 3d and contour plot of the respective surface, where emotional exhaustion is lowest when both closeness levels are low or high (lower-left corner as well as upper-right corner appear most reddish). The significant *a*_3_ coefficient (*a*_3_ = 1.73 *p* = 0.010) tells us that increasing incongruent relationship experiences in the direction of “most attached student becomes closer than the least attached student” are associated with increasing exhaustion. Similarly, the positive estimate of *a*_4_ (*a*_4_ = 0.55, *p* = 0.071) reveals that with increasing range of relationship quality, teachers are more emotionally drained and depleted. Accordingly, in the contour plot (2A) the color changes from green to reddish when following the LOIC to the upper-left corner.

Regarding Personal Accomplishment, the best model among the candidates was the null model. Within the range of ΔAICc < 2 were further SRSQD, SQD and RR models. All those models were statistically insignificant and revealed inacceptable model fit with CFI values up to 0.74. Thus, no model can be reported for personal accomplishment. In conclusion, student–teacher relationship range might not impact teachers’ personal accomplishment.

### Q2: Impact of Teacher’s Attachment Security and Relationship Closeness on Burnout

In order to test for the combined effect of attachment security (*X*-variable) and most attached student’s closeness (*Y*-variable), the same procedure as in Q1 was repeated. For Depersonalization, SSQD was the best model regarding AIC, followed by SRR, SQD, SRSQD, SRRR and RR models which were equally plausible due to ΔAICc < 2 (**Table 2**). We chose the SRR model, because it was more superior regarding model weight (AICcWt = 0.26) and evidence ratio and also showed acceptable model fit with a CFI > of 0.98. SSQD revealed an *R*^2^ of 0.159 and was overall significant (*p* < 0.001).

Investigating the fit hypothesis reveals a significant positive *a*_4_ coefficient (*a*_4_ = 1.13, *p* = 0.004), suggesting an upward curve along the line of incongruence (**Table 4**, **Figures 3A,B**). Thus, increasing relationship incongruence goes along with increased depersonalization. According to the contour plot in **Figure 3A**, the effect is most prominent when attachment security is higher than closeness of the most attached student, because highest depersonalization can be found here (most yellowish part in the right-upper corner). Results also suggest that no evidence for a mean-level effect was found.

**Table 4:** Response surface analysis (RSA) Coefficients Model 2: impact of attachment security and relationship closeness with student.

Model	Estimate	Robust *SE*	95% CI (lower)	95% CI (upper)	*p*
**Depersonalization – SSQD model**					
*b*_1_	0.154	0.085	-0.012	0.322	0.070
*b*_2_	-0.154	0.085	-0.322	0.012	0.070
*b*_3_	0.282	0.099	0.087	0.476	0.004
*b*_4_	-0.564	0.198	-0.953	-0.175	0.004
*b*_5_	0.282	0.099	0.087	0.476	0.004
*a*_4_	1.130	0.396	0.351	1.907	0.004
C	0.274	0.153	-0.027	0.575	0.074
**Emotional exhaustion – SQD model**					
*b*_1_	9.96e-10	2.83e -17	9.96e-10	9.96e-10	<0.001
*b*_2_	9.94e-10	4.88e -17	9.94e-10	9.94e-10	<0.001
*b*_3_	0.262	0.115	0.035	0.488	0.023
*b*_4_	-0.524	0.231	-0.977	-0.0704	0.024
*b*_5_	0.262	0.115	0.035	0.488	0.024
*a*_4_	1.048	0.4.62	0.140	1.950	0.024

*SSQD, shifted squared difference model; SQD, squared difference model.*

A similar image was obtained for Emotional Exhaustion. The best fitting model according to AIC was the SQD model. Equally plausible according to ΔAICc < 2 were the RR and null models. Because SQD showed higher model weight and evidence ratio and RR model was statistically insignificant (*p* = 0.103), we selected the SQD model. Relative model fit of SQD with a CFI of 1 was good. The SQD model explained 4.6% of the variance of emotional exhaustion, but due to the fact that the model statistically only tended toward significance (*p* = 0.051), parameters should be interpreted with caution. There is again no evidence for a mean-level effect.

The significant coefficient *a*_4_ (*a*_4_ = 1.048, *p* = 0.024) reveals that increasing discrepancy of both relationships predicts increased emotional exhaustion. Inspection of the contour plot in **Figures 4A,B** also reveals that emotional exhaustion increases with increasing incongruence as the surface gets more greenish toward the upper-left and lower-right corners.

Again, for Personal Accomplishment, no model can be reported because the best fitting model according to AIC was the null model and all other models were statistically insignificant.

## Discussion

The purpose of the current study was to investigate the links between teacher burnout and teachers’ relationship experiences. Particular attention has been paid to how the teacher’s extent of connectedness and relationship diversity with students are linked with burnout. Therefore, relationship closeness of teachers’ most and least attached students was assessed as well as attachment security. Furthermore, we aimed to understand how teachers’ connectedness toward students, in combination with teachers’ attachment security, affects teachers’ wellbeing.

One of the strengths of the current paper is the focus on the positive aspect of relational exchange instead of focusing on problematic student behavior, which has been more frequently focused on in teacher burnout research (Friedman, 1995; Hastings and Bham, 2003; Evers et al., 2004; Kokkinos, 2007; Chang, 2013). Thus, we follow the significant debate around positive psychology research (Seligman and Csikszentmihalyi, 2000) and provide a first attempt at showing that relationship closeness potentially enhances teachers’ wellbeing. Moreover, the RSA methodology used in order to investigate the combined effects of two variables on an outcome might further stimulate burnout as well as relationship research as it provides the opportunity to test for hypotheses focusing on congruence and incongruence. These are issues that both research areas are interested in.

With regard to the current findings, we found support that teachers’ connectedness with students plays a significant role regarding teachers’ burnout, whereas attachment experiences were found to play a minor role. As expected, teachers developing close relationships with their students felt less burned out than teachers who establish more distant and more incongruent relationships. In contrast to our expectation, teachers’ attachment experiences were not directly associated with teachers’ wellbeing, but seem to have a tendency to foster teacher’s capabilities to form close relationships with students.

### The Role of Teachers’ Relationships with Students, on Burnout

In more detail, RSA analyses revealed that depersonalization and emotional exhaustion were lowest when teachers developed homogenous high quality relationships toward their students within the classroom. Thereby, the current finding goes beyond research, which showed that relationships with students were reported to be rewarding and that lacking reward leads to burnout (Hargreaves, 2000; Unterbrink et al., 2007).

Even though the current study only measured emotions on a representational level, we assume within attachment framework that daily emotions guided by the representational models are activated by students’ daily behavior, as a previous review outlined in more detail (Spilt et al., 2011). This argument can be supported by research on secondary classroom teachers showing that relational closeness with students was strongly positively related to teachers’ emotional joy and negatively to teachers’ anxiety and anger (Hagenauer et al., 2015). Furthermore, the current findings can be seen to be in line with meta-analytic work, revealing that emotional responses predicted the level of burnout (Montgomery and Rupp, 2005) and thereby also positive teacher emotions such as enjoyment were a buffer for emotional exhaustion (Keller et al., 2014).

In order to explain the positive emotions that relationship closeness might evoke, we also rely on SDT which states that warm and open relationships might fulfill a teacher’s need of relatedness but also enhances a teacher’s feeling of competence and support; and thus might be protective for teachers’ wellbeing (Deci and Ryan, 2000; Hakanen et al., 2006; Ditzen and Heinrichs, 2014). The idea that affective work pays off because it enables teachers to be more effective within the learning processes could explain this further (Hargreaves, 2000). Accordingly, teachers’ enjoyment has been found to enhance students’ enjoyment within classrooms, mediated by teacher’s enthusiasm (Frenzel et al., 2009). Thus, high quality relationships might help in creating an atmosphere in which students as well as teachers feel motivated, respected and valued. This might reduce conflicts, potentially increase joyful learning opportunities and foster teachers’ feelings of effectiveness. This idea can be further supported by the fact that high quality relationships are associated with a range of positive outcomes and thus high quality relationships might also reflect a productive classroom which also triggers a teacher’s feelings of competence (Davis, 2003; Pianta et al., 2003; Milatz et al., 2014). As a consequence, teachers might also feel supported by their students and be effective in pursuing their goals, both relevant markers for wellbeing (Montgomery and Rupp, 2005; Chang, 2013; Ditzen and Heinrichs, 2014). In conclusion, it is important to note that the current study does not include those emotional variables. Thus, the above outlined arguments only provide an explanation as to why relational closeness would lead to lower burnout. Future studies should directly assess the hypothesized links.

Surprisingly, no RSA model investigating the combined effect of most and least attached student on personal accomplishment was significant. Thus, teachers’ relationship range does not affect their feelings of effectiveness. Instead, the most attached student’s closeness was associated with personal accomplishment, revealing that a single significant relationship potentially enhances teachers’ feelings of competence. This provides further evidence for the assumption within SDT that student–teacher relationships are expected to foster a teacher’s motivation and thus might be important resources in a teacher’s life (Hakanen et al., 2006).

Interestingly, teachers developing homogenous low quality relationships also reported an equally low degree of emotional exhaustion as teachers who developed high quality relationships. This seems counterintuitive at first glance but seems reasonable when considering that low connectedness toward students also means low relationship engagement, which in turn might be a strategy to save energy and to feel less exhausted. This would correspond with the work related withdrawal pattern, in which individuals are characterized by low ambition and a high ability to distance from others (Kieschke and Schaarschmidt, 2008). Whether an individual has the feeling that relationship investment pays off or not and accordingly invests or refuses to invest in relationships, might depend on how an individual judges, for example, importance, goal congruence and responsibility of relationships, as reflected in teachers’ implicit theories of their classroom relationships (Chang and Davis, 2009).

Further results suggest that a teacher’s relationship range also impacts their wellbeing, even though results only tended toward significance. Depersonalization and Emotional Exhaustion increased as relationship range increases, or in other words, as teachers make more incongruent relationship experiences with the students. As teachers might feel responsible for developing productive relationships with their students in order to support learning and development (O’Connor, 2008; Chang and Davis, 2009), relationship incongruence might interfere with a teacher’s goal to care equally for his/her students (Spilt et al., 2011). An increasing deviance of teacher and student goals was already shown to be associated with increased negative feelings, leading to burnout (Chang, 2013).

It can be hypothesized that the teachers’ actual experienced feelings toward students might not correspond with their expected or supposed feelings. Motive-incongruence has been shown to increase emotional labor, which in turn was argued and identified to be related to burnout (Johnson et al., 2005; O’Connor, 2008; Chang, 2013; Keller et al., 2014). Thus, increased emotional labor might be reflected in teachers’ incongruent relationship experiences and provides an explanation as to why those experiences are associated with poorer wellbeing. Incongruent experiences might also lead to mixed feelings regarding the need of relatedness and thus might evoke feelings of frustration about being unable to reach every student and not being supported by them. In contrast, individuals with rather congruent relationship experiences might have a higher match of relationship expectations and experiences with students and thus perceive it as goal congruent and fulfilling, potentially leading to positive emotions.

### The Role of Teachers’ Attachment and Connectedness on Burnout

The findings from the second set of RSA models investigating the combined effect of teachers’ attachment experiences and teachers’ connectedness with the most attached student also revealed that incongruent relationship experiences correlate with increased burnout. Most importantly, the plots revealed that burnout was lowest when attachment experiences were secure but relationship quality with the most attached student was only in the mid-range. In other words one could say, when teachers’ actual relationship quality with the most significant student is worse than the potential capability to establish close relationships, teachers reported a higher level of depersonalization and exhaustion.

Again, teachers may experience incongruent relationships through negative feelings, as the desired relationship goal as derived from own attachment security does not match with the actual relationship as experienced in relationships with students. This might cause increased emotional labor, which in turn affects a teacher’s wellbeing (Chang and Davis, 2009; Keller et al., 2014). The incongruence might also lead to a teacher’s need of relatedness not being fulfilled, as they have internalized that relationships can be even more rewarding and supportive. Teachers whose desired and actual relationship experiences are congruent might feel effective, competent and rewarded in terms of their relational work, which diminishes burnout, as explained previously (Skaalvik and Skaalvik, 2010).

Explanations of how the opposite incongruent relationship experience affects burnout seem to be less obvious. Perhaps teachers with closer relationship experiences with students than with their mothers might undergo a process of correcting relationship experiences as already suggested by Riley (2009), which could imagined to be a stressful personal growth experience. It is important to note that the results for emotional exhaustion need to be treated with caution as the overall model failed significance (*p* = 0.051) even though the main parameter *a*_4_ was significant.

No support could be found for the hypothesis that the highest burnout can be found for individuals low on attachment security and low on relational closeness with the most attached student. Furthermore, no support was found for the reversed effect that the lowest burnout appeared with high levels on both scales. An explanation could be that due to the relatively high attachment security and low burnout level of the current teacher sample, a respective pattern could not be found.

Attachment security could also not be directly associated with burnout. This finding is not in line with research showing links of attachment style with burnout, for example on a sample of security guards and university employees (Schirmer and Lopez, 2001; Vanheule and Declercq, 2009). It could be argued that primary attachment such as with one’s mother might not be that important within a teacher’s daily school life as there are other crucial interpersonal relations aside from with students such as with colleagues and administrators (Skaalvik and Skaalvik, 2010). Even though further research is required, this would be a positive message with respect to its practical consequences because supporting teachers to develop more close relationships toward their students might be more promising than getting teachers to reflect on their attachment representations.

As expected, attachment has been shown to be relevant for teachers’ ability to establish close relationships at least toward the most attached student. More secure attachment experiences lead to a higher connectedness. Thus, this evidence is in line with research and further extends to the finding based on a pre-service teacher sample (Kesner, 2000). Findings are also concordant with adult attachment research showing that secure individuals show higher socio-emotional competences than their insecure counterparts (Priel and Shamai, 1995; Mikulincer and Florian, 1997; Mikulincer et al., 2001; Collins and Feeney, 2004; Mikulincer and Shaver, 2007; Mikulincer et al., 2009). Since we cannot test any causal links with the current data, the current findings might also stimulate research on student–teacher relationships as it points out teacher characteristics, which might also be potentially important in the establishment of student–teacher relationships.

Most importantly, we contributed to research of teacher burnout, which, until now, falls short in addressing interpersonal needs and emotions as embedded in teachers’ relationships with students. All in all, we provide evidence for the assumptions that teachers’ diverse emotions and cognitions as reflected in relationship closeness with their students are a source of teachers’ wellbeing (Zembylas and Schutz, 2009; Spilt et al., 2011; Chang, 2013).

### Limitations and Future Research

Three limitations of this study should be noted. First, the study falls short of any direct emotion measurement in addition to the relationship representations. Therefore, we do not fully understand how actual experienced emotions may trigger wellbeing. Further research should include teachers’ positive and negative emotional experiences (e.g., enjoyment, anxiety, anger) in the classroom (Keller et al., 2014) as well as more student behavior measures (e.g., discipline, insults, positive feedback; Unterbrink et al., 2008). This would allow for a full assessment of the assumptions made by Spilt et al. (2011).

Second, it is important to note that the study is based on a cross-sectional design and no causal interpretations can be made. Thus, effects might also work the other way around and burnout might influence how teachers establish relationships. In order to empirically distinguish between effects and consequences, further longitudinal studies are required to assess the extent to which relationships positively affect burnout or whether relationship experiences are rather a mirror of the teachers’ wellbeing.

Even though prior research showed teachers’ ratings of relational closeness as captured with the STRS to be valid as it is reflected in external observations and student reports (Milatz et al., 2014), future research should include multiple perspectives (including students) and multiple methods such as teacher interviews or classroom observations (objective classroom variables) in order to validate findings based on teachers’ self-report perceptions.

Third, it needs to be mentioned that measuring teachers’ relationship range does not fully reflect a teacher’s total relationship experiences in class. Thus, the current approach is just a first attempt at looking at those questions of relationship diversity and congruent/incongruent relationship experiences. It needs to be validated in further studies as to whether relationship range is a good measure to assess the relationship variety of a teacher.

From the methodological perspective, the practical value derived from this study lies in the application of the RSA models. Those models seem to be meaningful, as the combination of two predictors of an outcome can be tested with mean-level and incongruence/congruence effects (Shanock et al., 2010; Schönbrodt, 2015a). As burnout research covers questions of imbalance of needs/desires and actual experiences, it would be a crucial tool for investigating how levels and discrepancies lead to higher or lower levels of burnout. This approach would also be promising for attachment or relationship research as relationship congruence questions are heavily discussed. As a methodological limitation, it should be noted that those RSA models currently do not allow for the incorporation of covariates, such as, for example, the demographic characteristics of the teachers.

Despite these limitations and possible future research, the current paper provides empirical evidence that student-teacher relationships can be seen as an important resource in a teacher’s daily life. The perspective that student–teacher relationships are important for teachers as well as for students might also motivate practitioners to enhance relationship quality and to detect possible sources of meaningful change. For example, the intervention program Banking Time, designed to enhance relational quality between a teacher and a preschooler might provide evidence-based practical suggestions as to how congruent close relationships in the classroom can be fostered (Williford et al., 2015). This intervention program focuses on secluded teacher–child interaction time in which the teacher is asked (1) to observe the child’s behavior and emotions, (2) to narrate the child’s actions and to follow their intentions, (3) to label the child’s emotions in order to understand the child’s perspective and (4) to incorporate relevant relational themes. This knowledge should strengthen burnout intervention or rehabilitation programs as already successfully implemented in a group based coaching program (Unterbrink et al., 2011). Among others, one focus of this program entails establishing trustful relationships toward students by sensitizing teachers for (1) being aware of the students and their perspectives, (2) being authentic as a person, (3) engaging in joint attention and action and (4) being empathetic. In sum, the current study acknowledges once again the importance of teachers’ relational work and stresses that teachers require time and resources to work on their relationships with students.

## Author Contributions

All listed authors contributed meaningfully to the paper as they all fulfilled the four requirements as stated in “Frontiers of Psychology” (http://journal.frontiersin.org/journal/psychology#author-guidelines): substantial contributions to the conception or design of the work; or the acquisition, analysis, or interpretation of data for the work; AND Drafting the work or revising it critically for important intellectual content; AND Final approval of the version to be published; AND Agreement to be accountable for all aspects of the work in ensuring that questions related to the accuracy or integrity of any part of the work are appropriately investigated and resolved.

## Conflict of Interest Statement

The authors declare that the research was conducted in the absence of any commercial or financial relationships that could be construed as a potential conflict of interest.

## Footnotes

1 Teachers’ relationship quality with the most attached (*Y*) and least attached (*X*) students closeness is a special case in which only one direction is given by default (*Y* > *X*).
